# Isolated fallopian tube torsion (IFTT) associated with hydrosalpinx in an adolescent: a case report

**DOI:** 10.1097/RC9.0000000000000713

**Published:** 2026-07-15

**Authors:** Majd Oweidat, Haya Taha, Abd Alqader Alwohoush, Yousef Albow, Islam Alshawawra, Ammar W.M. Hassouneh

**Affiliations:** a College of Medicine, Hebron University, Hebron, West Bank, Palestine; b Palestine Red Crescent Specialized Hospital - Hebron, Hebron, West Bank, Palestine; c Department of Radiology, Princess Alia Hebron Governmental Hospital, Hebron, West Bank, Palestine; d Faculty of Medicine, Al-Quds University, Jerusalem, West Bank, Palestine; e Department of Radiology, Al-Mezan Specialty Hospital, Hebron, West Bank, Palestine

## Abstract

**Introduction:**

Isolated fallopian tube torsion (IFTT) is a rare and often under-recognized cause of acute lower abdominal pain, particularly in adolescents. Clinical features are nonspecific and frequently mimic more common conditions, such as appendicitis or ovarian torsion, leading to delayed diagnosis and an increased risk of tubal necrosis. Association with hydrosalpinx in young patients without traditional risk factors is uncommon.

**Case presentation:**

A 15-year-old nulligravid girl with regular menses and no history of sexual activity or prior pelvic surgery presented with a 2-day history of severe, continuous lower abdominal pain, nausea, and vomiting. Examination revealed localized lower abdominal tenderness without peritoneal signs. Laboratory tests showed leukocytosis with neutrophilia; urinalysis was normal. Transabdominal ultrasonography was inconclusive due to body habitus. Contrast-enhanced computed tomography showed a right-sided cystic tubular adnexal structure consistent with hydrosalpinx, a central whirlpool configuration, a normal right ovary, and minimal free fluid, raising suspicion for IFTT. Urgent laparotomy confirmed isolated torsion of the right fallopian tube with necrosis; right salpingectomy was performed. The postoperative course was uneventful.

**Discussion:**

This case highlights the diagnostic challenge of IFTT. The coexistence of hydrosalpinx in an adolescent without prior infection or surgery underscores a possible role of tubal pathology in the pathogenesis of torsion.

**Conclusion:**

IFTT should be considered in adolescents with unilateral pelvic pain and normal-appearing ovaries to enable timely, fertility-conscious management.

## Introduction

The work has been reported in line with the SCARE Guidelines 2025 criteria^[^1^]^.

Isolated fallopian tube torsion (IFTT) is a rare and often overlooked cause of acute lower abdominal pain. It occurs when the fallopian tube twists around its own axis while the ipsilateral ovary remains uninvolved, which can compromise venous and lymphatic outflow, produce pelvic congestion, and ultimately result in ischemic damage to the tube^[^2^]^. The condition appears to affect the right side more commonly, possibly because of anatomical factors such as stabilization of the left tube by the sigmoid colon. Although IFTT can occur at any age, it is uncommon overall, with an estimated prevalence of roughly 1 in 1.5 million women, and it is reported even less frequently in children and adolescents^[^2,3^]^.

HIGHLIGHTS

- Preoperative CT raised a strong suspicion of isolated tubal torsion.
- Hydrosalpinx occurred in a teenager without prior surgery or infection.
- Normal-appearing ovary does not exclude significant tubal pathology.
- Early recognition supported timely surgery with fertility-focused planning.

Clinical recognition is difficult because symptoms are nonspecific and overlap with more common diagnoses, including appendicitis and ovarian torsion. As a result, many cases are only identified intraoperatively, and delays in treatment increase the likelihood of necrosis, irreversible tubal injury, and infectious complications^[^2^]^.

In this report, we describe a case of IFTT associated with hydrosalpinx, which was suspected preoperatively on imaging and confirmed surgically.

## Case presentation

A 15-year-old female patient, nulligravid, with regular monthly menstrual cycles since menarche in early adolescence and no history of sexual activity, presented to the emergency department with a 2-day history of sudden, severe lower abdominal pain. She described the pain as sharp, constant, progressively worsening, radiating to the back, and rated it as maximal severity. It was accompanied by nausea and repeated vomiting, with no history of fever, and it did not improve with oral analgesics. Her last menstrual period had occurred approximately 1 month earlier, with no preceding cycle abnormalities, intermenstrual bleeding, or dysmenorrhea. She denied any vaginal discharge, urinary symptoms, bowel changes, history of trauma, or prior episodes of similar pain. Her past gynecologic history was unremarkable, with no prior surgeries and no history suggestive of sexually transmitted infections. She reported no use of medications, no allergies, and no exposure to tobacco or other substances. Her family and social history were unremarkable.

On arrival, her temperature was 37.5 °C; she was hemodynamically stable, with normal oxygen saturation and a body mass index of 34.6 kg/m^2^. She appeared uncomfortable but alert. Abdominal examination showed localized tenderness in the lower abdomen without guarding or rebound tenderness, and bowel sounds were preserved. There were no palpable masses. A pelvic examination was declined due to the absence of sexual activity and the patient’s discomfort, except for the external inspection, which revealed no vulvar abnormalities or vaginal bleeding. A full systemic review identified no respiratory, cardiac, urinary, or gastrointestinal red flags beyond the presenting pain and vomiting.

**Figure 1.**
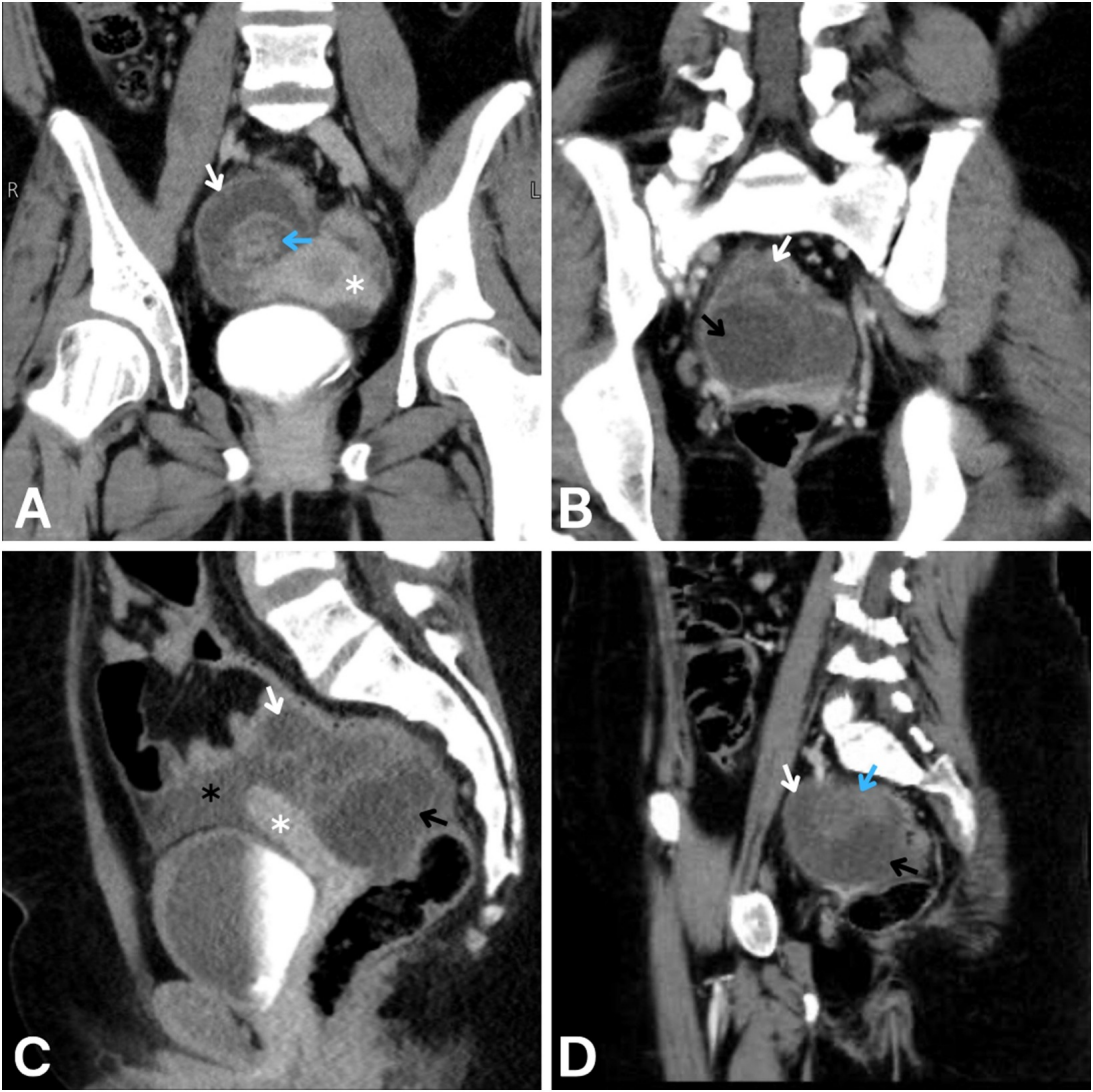
Contrast-enhanced abdominal CT showing the adnexal lesion in coronal (A and B), sagittal (C), and oblique (D) planes. (A) The upper segment of the hydrosalpinx (white arrow) is seen above the whirlpool sign of the twisted pedicle (blue arrow). The uterus is also visible (white asterisk). (B) Both the lower segment of the hydrosalpinx (black arrow) and the upper segment (white arrow) are shown. (C) Sagittal plane showing the spatial relationship between the lower segment of the hydrosalpinx (black arrow), the upper segment (white arrow), and the uterus (white asterisk). A small amount of free fluid is present (black asterisk). (D) Oblique view showing continuity between the two hydrosalpinx segments (white and black arrows), with the whirlpool sign centrally located (blue arrow).

Initial laboratory evaluation showed leukocytosis with neutrophilia and an elevated platelet count, while urinalysis was normal. These findings suggested an acute inflammatory process but were nonspecific regarding its source. Erythrocyte sedimentation rate (ESR) and C-reactive protein (CRP) were within the normal ranges. The first-line abdominal ultrasound (US) failed to visualize the adnexa due to body habitus, though the uterus appeared normal; this prompted further imaging. An abdominopelvic computed tomography (CT) scan, both without and with iodinated contrast, revealed a cystic tubular structure in the right adnexa consistent with hydrosalpinx, accompanied by a central whirlpool configuration near the origin of the fallopian tube (Fig. 1). The right ovary showed normal size and enhancement, and a small volume of free pelvic fluid was noted. The imaging pattern, combined with the abrupt, persistent pain and lack of ovarian enlargement, raised concern for IFTT. Given the risk of irreversible ischemic damage, the decision was made to proceed with urgent surgical exploration.

Under sterile conditions and general anesthesia, the patient underwent laparotomy. After entering the peritoneal cavity through sequential layer dissection, the right fallopian tube was found to be twisted, with a segment showing dark discoloration consistent with necrosis and markedly edematous fimbriae. Both ovaries appeared healthy and well-perfused. Because the devitalized segment of the right fallopian tube didn’t regain its normal color sufficiently after untwisting, a right salpingectomy was performed, preserving the contralateral adnexa. The excised fallopian tube was sent for histopathological examination. No additional pelvic or abdominal abnormalities or congenital anatomic variants were identified. The abdomen was closed in layers, and the patient recovered without intraoperative complications.

On histopathological examination, the specimen measured 7 × 3 × 3 cm. Microscopic examination confirmed hydrosalpinx with a dilated tubal lumen and flattened mucosal plicae, along with hemorrhage and hemorrhagic necrosis of the tubal wall. Only scattered residual ciliated cells were identified in the fimbrial region, and no evidence of malignancy was found.

Postoperatively, she showed rapid clinical improvement and tolerated oral intake. She was discharged after 24 hours on oral analgesic treatment, with instructions to keep the wound clean and dry, gradually resume activity as tolerated, and seek urgent medical care if fever, worsening abdominal pain, wound redness, bleeding, discharge, or any other concerning symptoms developed. At the 1-week postoperative review, she reported complete resolution of pain, normal mobility, and no fever, bleeding, discharge, or wound-related concerns. The incision was clean and healing appropriately. She was advised to return earlier if any symptoms or complications occurred and was followed regularly at 2 months, 6 months, and 1 year. At the latest follow-up, she remained asymptomatic with no evidence of postoperative complications. Follow-up transabdominal US showed normal-appearing ovaries, with no recurrent adnexal cysts and no pelvic free fluid.

## Discussion

This report describes an adolescent girl with isolated torsion of the right fallopian tube in association with hydrosalpinx, in whom radiologic findings prompted early suspicion and urgent surgical intervention. Although the condition is anatomically confined to the tube, the clinical picture closely resembles more common causes of acute abdomen, and this diagnostic ambiguity often delays treatment. Our case highlights the importance of considering IFTT in adolescents presenting with severe, persistent lower abdominal pain, particularly when imaging reveals a dilated tubular adnexal structure with preservation of ovarian morphology.

IFTT is regarded as a rare gynecologic emergency^[^2^]^. Further complicating recognition, the etiologic mechanisms of tubal torsion remain incompletely understood. Twisting typically occurs around the axis formed by the mesosalpinx and tubo-ovarian ligament, provoking venous congestion and progressive ischemia. The ovary is often spared due to its dual arterial supply, which helps explain why normal ovarian appearance on imaging does not exclude pathology in the tube^[^2^]^.

Multiple predisposing factors have been proposed in adults, including hydrosalpinx, post-surgical adhesions, paraovarian cysts, prior pelvic inflammatory disease, and endometriosis^[^4^]^. In children and adolescents, however, clear risk factors are less well-defined. Congenital anatomic variants, such as elongated mesosalpinx or developmental tubal irregularities, have been hypothesized, and puberty-related hormonal influences may also alter tubal motility and elasticity^[^2^]^. Hydrosalpinx itself is an unusual finding in adolescents without prior infection or surgery, raising ongoing debate about whether it precipitates torsion or represents a secondary consequence of the twisting process^[^5^]^. In our case, the radiologic impression of hydrosalpinx alongside torsion invites both possibilities.

Clinically, IFTT classically presents with abrupt lower abdominal pain, often accompanied by nausea and vomiting. Fever tends to occur late, typically when ischemia advances or secondary infection develops^[^6^]^. In adolescent females, acute right lower quadrant pain has a broad differential diagnosis, and IFTT may be overlooked because it clinically overlaps with more common gastrointestinal, urinary, and gynecologic emergencies. Important alternatives include acute appendicitis, ovarian torsion, ruptured or hemorrhagic ovarian cyst, ectopic pregnancy in patients with pregnancy potential, pelvic inflammatory disease or tubo-ovarian abscess, ureteric colic, pyelonephritis, incarcerated hernia, and bowel obstruction^[^7^]^. Therefore, evaluation should integrate menstrual and sexual history, urinary and gastrointestinal symptoms, inflammatory markers, pregnancy testing when applicable, and targeted imaging.

Imaging plays an important, though imperfect, role. US remains the first-line modality in adolescents because it is widely available and avoids radiation. Hallmark sonographic features include a dilated, fluid-filled tubular structure tapering toward the uterine cornua, separate from a normal-appearing ovary. The “whirlpool sign,” representing a twisted vascular pedicle, is highly suggestive, yet it is not always visualized, and Doppler flow may persist despite torsion^[^8,9^]^. CT and magnetic resonance imaging can reveal additional clues, including a distended tube, paraovarian cysts, pelvic fluid, or a spiral configuration of mesosalpinx vessels. However, literature shows that preoperative diagnostic accuracy remains low, with many cases diagnosed definitively only during surgical exploration^[^2,8,9^]^. In this context, our case is notable because CT features strongly supported IFTT preoperatively, showing growing evidence that careful interpretation of cross-sectional imaging can guide earlier intervention.

Management strategies aim to preserve fertility whenever feasible while preventing complications such as necrosis, hemorrhage, infection, or recurrent torsion. Prompt laparoscopy is generally favored as the diagnostic and therapeutic gold standard. When the tube appears viable after detorsion, conservative approaches, including neosalpingostomy or cyst excision, may be appropriate, particularly in adolescents^[^5^]^. Nevertheless, surgeons must balance the desire for conservation with the reality that severely ischemic or necrotic tubes pose risks and rarely regain function^[^5^]^. Literature suggests that many pediatric cases ultimately undergo salpingectomy because the tube fails to reperfuse or is structurally compromised^[^4,5,10^]^. In addition, symptom duration appears to influence surgical outcomes, as pediatric IFTT cases presenting after more than 24 hours have been reported to have a significantly higher likelihood of salpingectomy compared with cases treated earlier^[^2^]^. Our patient had symptoms for 2 days before presentation, which may have contributed to the irreversible ischemic changes observed intraoperatively.

The literature remains divided on how aggressively to pursue conservative tubal preservation. Some authors report acceptable outcomes following staged procedures, with second-look laparoscopy used to reassess tubal viability and potentially reconstruct the fimbriated end. Others caution that preserved, yet dysfunctional, tubes may increase later risks of ectopic pregnancy or require repeat surgery^[^5,8,9^]^. There is no uniform guideline, and decisions must be individualized based on intraoperative appearance, patient age, reproductive goals, and surgeon expertise.

From an educational perspective, our case contributes in several ways. First, it highlights an unusual diagnostic course in which imaging, rather than intraoperative discovery, raised early suspicion for IFTT. Second, it emphasizes the association between hydrosalpinx and tubal torsion in a young patient without typical risk factors, adding to the evolving discussion about pathogenesis. Third, it shows how delayed recognition risks irreversible tubal damage and highlights the importance of including IFTT in the differential diagnosis of adolescent acute abdomen. Recognizing the imaging pattern of a tubular cystic structure with a normal ipsilateral ovary may prompt earlier gynecologic consultation and expedite surgery–a change in diagnostic thinking that carries clear implications for fertility preservation.

This report has inherent limitations as a single case, limiting generalizability and preventing causal conclusions regarding the relationship between hydrosalpinx and torsion.

## Conclusion

In adolescents with persistent unilateral pelvic pain and normal-appearing ovaries, a dilated tubular adnexal lesion with a whirlpool sign should prompt consideration of IFTT and an urgent gynecologic evaluation.

## Data Availability

The data supporting the findings of this case report are included within the article. No additional datasets were generated or analyzed during the current study.
